# Epilepsy in Duchenne and Becker muscular dystrophies

**DOI:** 10.1002/acn3.52058

**Published:** 2024-05-01

**Authors:** Jesus Alfonso Armijo Gómez, Miguel A. Fernandez‐Garcia, Ana Camacho, Marlin Liz, Carlos Ortez, Miguel Lafuente‐Hidalgo, Laura Toledo Bravo‐de Laguna, Berta Estévez‐Arias, Laura Carrera‐García, Jessica Expósito‐Escudero, Jana Domínguez‐Carral, Andres Nascimento, Daniel Natera‐de Benito

**Affiliations:** ^1^ Neuromuscular Unit, Department of Neurology Hospital Sant Joan de Déu Barcelona Spain; ^2^ Neuromuscular Unit Hospital La Paz Madrid Spain; ^3^ Division of Pediatric Neurology, Department of Neurology Hospital Universitario 12 de Octubre, Universidad Complutense de Madrid Madrid Spain; ^4^ Epilepsy Unit, Department of Neurology Hospital Sant Joan de Déu Barcelona Spain; ^5^ Applied Research in Neuromuscular Diseases Institut de Recerca Sant Joan de Déu Barcelona Spain; ^6^ Center for Biomedical Research Network on Rare Diseases (CIBERER), ISCIII Madrid Spain; ^7^ Department of Pediatrics Hospital Miguel Servet Zaragoza Spain; ^8^ Department of Pediatrics Hospital Materno‐Infantil Las Palmas de Gran Canaria Spain; ^9^ Laboratory of Neurogenetics and Molecular Medicine – IPER Institut de Recerca Sant Joan de Déu Barcelona Spain

## Abstract

**Objective:**

Duchenne and Becker muscular dystrophies (DMD and BMD) are dystrophinopathies caused by variants in *DMD* gene, resulting in reduced or absent dystrophin. These conditions, characterized by muscle weakness, also manifest central nervous system (CNS) comorbidities due to dystrophin expression in the CNS. Prior studies have indicated a higher prevalence of epilepsy in individuals with dystrophinopathy compared to the general population. Our research aimed to investigate epilepsy prevalence in dystrophinopathies and characterize associated electroencephalograms (EEGs) and seizures.

**Methods:**

We reviewed 416 individuals with dystrophinopathy, followed up at three centers between 2010 and 2023, to investigate the lifetime epilepsy prevalence and characterize EEGs and seizures in those individuals diagnosed with epilepsy. Associations between epilepsy and type of dystrophinopathy, genotype, and cognitive involvement were studied.

**Results:**

Our study revealed a higher epilepsy prevalence than the general population (1.4%; 95% confidence interval: 0.7–3.2%), but notably lower than previously reported in smaller dystrophinopathy cohorts. No significant differences were found in epilepsy prevalence between DMD and BMD or based on underlying genotypes. Cognitive impairment was not found to be linked to higher epilepsy rates. The most prevalent epilepsy types in dystrophinopathies resembled those observed in the broader pediatric population, with most individuals effectively controlled through monotherapy.

**Interpretation:**

The actual epilepsy prevalence in dystrophinopathies may be markedly lower than previously estimated, possibly half or even less. Our study provides valuable insights into the epilepsy landscape in individuals with dystrophinopathy, impacting medical care, especially for those with concurrent epilepsy.

## Introduction

Duchenne muscular dystrophy (DMD) and Becker muscular dystrophy (BMD) are dystrophinopathies, which are genetic disorders resulting from pathogenic variants in the *DMD* gene located on the X chromosome. These variants lead to a deficiency or absence of the essential protein dystrophin, crucial for maintaining muscle fiber integrity. In DMD, there is typically an absolute or nearly complete absence of dystrophin, whereas in BMD, the deficiency is typically partial. While DMD presents with severe and progressive muscle weakness and degeneration, BMD typically exhibits a milder clinical course.^1^, ^2^

Remarkably, dystrophin is not solely expressed in muscles but also in the brain, with both full‐length (Dp427c and Dp427p) and short (Dp140 and Dp71) isoforms present.^1^ These isoforms play critical roles in various brain regions, such as the hippocampus, cerebellum's Purkinje cells, and the amygdala, with implications in GABAergic and glutaminergic transmission, fetal development, and blood–brain barrier formation. Depending on the pathogenic variant site, specific isoforms may be affected, potentially leading to associations with neuropsychiatric and neurological disorders.^3^, ^4^, ^5^ However, it remains unclear whether the location of the pathogenic variant and the specific isoforms involved are directly associated with the development of epilepsy.

Despite the clinical significance of epilepsy in dystrophinopathies, the prevalence and characteristics of epilepsy in this population have not been thoroughly studied, resulting in varying estimates in existing literature (ranging from 3.1% to 7.9%).^6^, ^7^, ^8^, ^9^, ^10^, ^11^ With increasing interest in therapies aiming to improve motor outcomes in individuals with dystrophinopathies, it becomes also relevant to explore the impact of extramuscular involvement, as it is not expected to be amenable to modification through these emerging therapeutic approaches.^12^, ^13^

In light of this knowledge gap, our study aims to investigate the lifetime prevalence, onset, and evolution of epilepsy in a cohort of 416 individuals with dystrophinopathy, closely followed over an extended period. We strive to provide a comprehensive analysis of seizure and epilepsy types, electroencephalographic (EEG) features, and the response to antiepileptic therapies, aiming to gain valuable insights into the impact of epilepsy within this context. Furthermore, we aim to establish potential correlations between the epileptic phenotype and various clinical factors, including the motor function, the cognitive involvement, and the genetic background.

## Methods

### Study design and inclusion criteria

This retrospective study was performed on data collected in the neuromuscular units of three centers: Hospital Sant Joan de Déu, Barcelona; Hospital 12 de Octubre, Madrid; and Hospital La Paz, Madrid. To address potential sources of selection bias, we included all individuals with diagnosis of dystrophinopathy who were under follow‐up at these three centers between 2010 and 2023.

Despite its retrospective nature, subsequent to the compilation of individual data from medical records, the assessment of EEGs and seizure types underwent meticulous review specifically for this study. Information regarding seizures and epilepsy extracted from medical records was validated through discussions with families concerning seizure characteristics and a thorough examination of available EEG records.

### Phenotyping

We reviewed the medical records of the 416 individuals with dystrophinopathy followed up at the three centers to investigate the lifetime prevalence of epilepsy and to provide a characterization of the EEGs and seizures in those individuals diagnosed with epilepsy. Our analysis encompassed a thorough examination of clinical, electroencephalographic (EEG), and genetic data. Individuals were classified as having Duchenne or Becker muscular dystrophy based on clinical criteria, ensuring alignment between phenotype and functional motor scale results. Additionally, we verified that none of the individuals categorized as having Becker muscular dystrophy had experienced loss of ambulation before the age of 20 years.

Neuropsychological assessments were available for a subset of participants, not universally accessible. For the purposes of this study, an individual was categorized as having intellectual disability if their intellectual quotient measured below 70, or if explicitly noted by the neurologist in their report. Moreover, an individual was considered to have “poor academic performance” if he had repeated a grade or required significant curricular adaptations.

Epileptologist JND specifically reviewed the available EEG records of individuals with seizures for this study. EEGs had been performed in accordance with the international standard 10–20 system, utilizing Ag/AgCl electrodes. The polygraphic signals included ECG, surface EMG, and pneumogram, and they were recorded using computerized systems. Our evaluation covered the EEG background activity during both wakefulness and sleep, as well as the identification, morphology, localization, and persistence of any epileptiform anomalies, adhering to the International Guidelines of Neurophysiology.^14^, ^15^ To ensure the accuracy of seizure and epilepsy classification for each individual, the characteristics of seizures and EEGs were specifically reassessed for this study. This process included contacting families of individuals with epilepsy to gather comprehensive information, thereby minimizing potential errors or gaps in medical histories.

Seizures and epilepsy were described and classified according to the criteria set forth by the International League Against Epilepsy (ILAE) classification.^16^, ^17^ The course of epilepsy was classified as (A) “early seizure freedom” (seizure‐free within 6 months of starting treatment), (B) “delayed seizure freedom” (seizures not immediately controlled by medication, but became seizure‐free at some point after 6 months), (C) “fluctuating course” (periods of seizure freedom of >12 months, interspersed with relapses), or (D) “refractory” (never seizure‐free for a continuous 12‐month period) following the method defined by Brodie et al.^18^

To assess the prevalence of epilepsy, we calculated both lifetime prevalence and active epilepsy prevalence. Lifetime prevalence is a form of period prevalence, which considers the period as the time between birth and the most recent assessment.

### Data of DMD variants

Similarly to other studies that categorize DMD variants based on their predicted impact on dystrophin isoform expression,^19^ individuals with dystrophinopathy were categorized into three distinct groups: Group 1 (*n* = 191) consisted of individuals with variants resulting in the absence of Dp427 while still maintaining the presence of Dp140 and Dp71; Group 2 (*n* = 54) consisted of individuals with variants resulting in the absence of Dp427 and Dp140 but retained Dp71; and Group 3 (*n* = 17) consisted of individuals with variants causing the absence of all three dystrophin isoforms‐ Dp427, Dp140, and Dp71. Individuals with variants exclusively involving the region upstream of intron 44 were classified as Dp427 negative and Dp140/Dp71 positive, placing them in Group 1. Individuals with variants involving exon 51 through exon 62, without affecting exon 63 or regions downstream of it, were categorized as Dp427/Dp140 negative but Dp71 positive, making them members of Group 2. Lastly, individuals with DMD variants affecting exon 63 or the regions downstream of exon 63 were classified as Dp427/Dp140/Dp71 negative, belonging to Group 3.^20^, ^21^ Individuals with DMD variants involving exon 45 to exon 50, excluding the region of exon 51 and downstream of it, were excluded from the analysis due to challenges in in predicting their effects on Dp140 expression, given that the Dp140 promoter is in intron 44 and its translation start site is in exon 51.^20^, ^21^

### Statistical analysis

Demographic and clinical characteristics were summarized using mean, standard deviation (SD), and range for continuous variables, and frequencies (percentage) for categorical variable. Correlation of the variables was established by Student's t‐test and, where applicable for continuous variables, Spearman's correlation coefficient. Values of *p*‐value <0.05 were considered statistically significant.

## Results

### Demographics and clinical features

**Figure 1:**
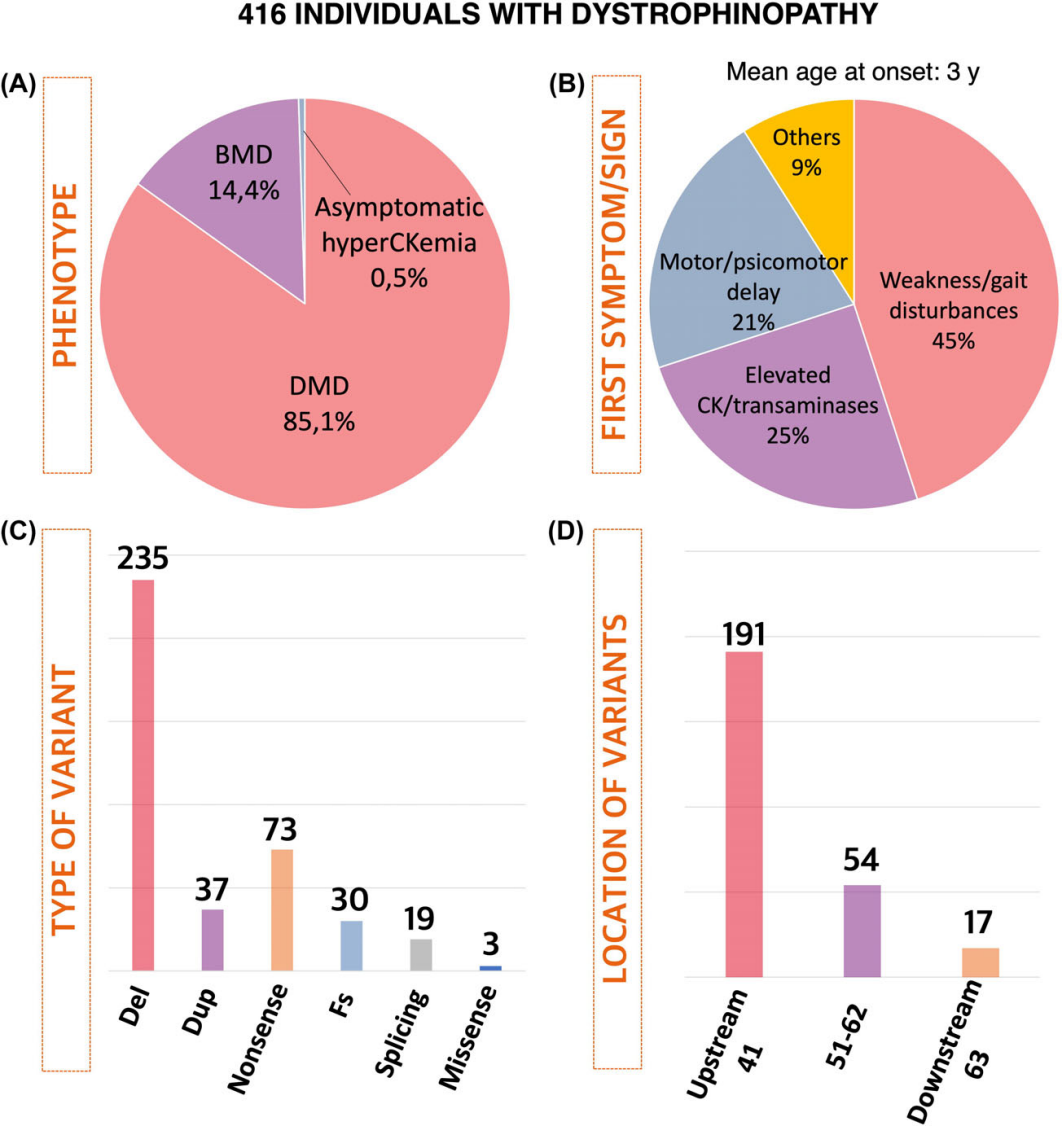
Pie charts show the distribution of phenotypes (A) and symptoms at onset (B). Bar charts illustrate the causative type of variants (C) and their location (D).

In this study, 416 individuals diagnosed with dystrophinopathy were included. Of these individuals, 85% had Duchenne muscular dystrophy, 14.5% had Becker muscular dystrophy, and 0.5% had asymptomatic hyperCKemia (Fig. 1). The mean age at the time of the most recent assessment was 12.4 years, with ages ranging from 1 to 24 years. The patient‐years of follow‐up were 5158.

Initial symptoms were documented at an average age of 3.0 years. The most frequently noted symptoms at the onset encompassed were as follows: (1) weakness/gait disturbances/frequent falls/difficulty rising from the floor (45.5%), (2) elevated levels of creatin kinase or transaminases in the blood (24.9%), (3) motor or psychomotor delay (20.8%), (4) myalgia or exercise intolerance (2.7%), and (5) a familial history of dystrophinopathy (2.5%) (Fig. 1). Among individuals who had lost the walking ability, the mean age of this occurrence was 11.2 years (SD: 2.98, range: 7–16 years). Intellectual disability and/or poor academic performance was observed in 24% of individuals (98/402).

### Genetic findings

The most prevalent type of genetic cause was deletion (59%), followed by nonsense variants (18%), duplications (9%), frameshift (8%), and splicing variants (5%) (Fig. 1). A total of 17 individuals had variants affecting all the brain isoforms of dystrophin (downstream of exon 63), 54 individuals had variants between exons 51 and 62, predicted to affect Dp427 and Dp140 expression but retaining Dp71, and 191 individuals had variants were located upstream of intron 44, resulting in the absence of Dp427 but maintaining Dp140 and Dp71 (Fig. 1).

### Prevalence of epilepsy and correlations

Epilepsy occurred in 6 of 416 individuals with dystrophinopathy (1.4; 95% confidence interval: 0.7–3.2%). Additionally, three individuals had typical febrile seizures (0.72%; 3/416): two had a single episode at 17 months and 3 years old, respectively, while one individual had two episodes at 2 and 4 years. Upon further categorization, the lifetime prevalence of epilepsy was determined to be 1.11% (4/354) among those with Duchenne muscular dystrophy and 3.33% (2/60) among individuals with Becker muscular dystrophy (*p* = 0.41). The mean age for losing the walking ability was comparable between individuals with epilepsy and those without it (11.2 years vs 11.0 years; p:0.89).

When investigating the occurrence of epilepsy in relation to cognitive involvement, it was observed that 0.99% (3/304) of those without cognitive impairment experienced epilepsy, as opposed to 3.1% (3/98) of those with intellectual disability or autism spectrum disorder who experienced epilepsy (*p* = 0.14).

Our analysis revealed a statistically significant relationship between the location of variants and the presence of intellectual disability or poor academic performance (p < 0.001). Notably, individuals with DMD variants affecting all the brain isoforms of dystrophin were significantly more likely to have intellectual disability or poor academic performance compared to other groups (p < 0.001). Cognitive involvement was observed in 73% (11/15) of individuals of Group 3 (variants downstream of exon 63, impacting all brain isoforms), compared with 27% (14/52) in Group 2 (variants in exons 51–62 affecting Dp427 and Dp140 expression) and 20% (38/186) in Group 1 (variants upstream of intron 44, only resulting in the absence of Dp427).

On the other hand, we did not find a significant association between genotype and the occurrence of epilepsy. When comparing subgroups with different variant types, the proportion of individuals with epilepsy was not significantly distinct: deletion (1.3%; 3/235), duplication (0%; 0/37), nonsense (0%; 0/73), and frameshift (3.4%; 1/29). Additionally, no statistically significant differences (*p* = 0.23) were found when comparing the location of the variants. Among individuals in Group 3 (variants downstream of exon 63 affecting all brain isoforms), 1 out of 17 had epilepsy (5.9%). None of the 54 individuals in Group 2 (variants in exons 51–62 affecting Dp427 and Dp140 expression) had epilepsy (0%), while 3 out of 191 individuals in Group 1 (variants upstream of intron 44 resulting in the absence of Dp427) had epilepsy (1.6%).

### Characterization of seizures and epilepsy

**Figure 2:**
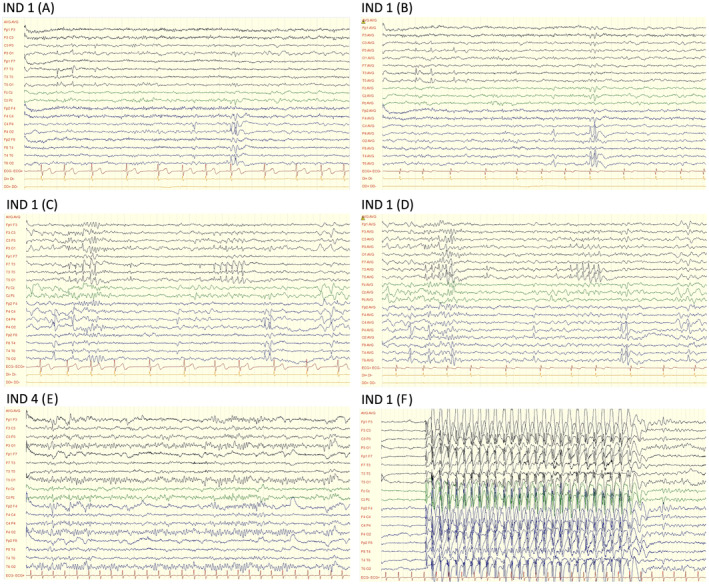
EEG studies of individuals 1 (A–D) and 4 (E and F). Individual 1: EEG showing typical findings in SeLECTS. Bipolar longitudinal (A) and monopolar average (B) montages during wakefulness, 10 sec per page, amplitude 100 mcV. Normal background activity with asynchronous spikes in left centrotemporal (C3‐T3‐T5) and right centro‐parieto‐temporal (C4‐P4‐T4‐T4‐T6) regions. Bipolar longitudinal (C) and monopolar average (D) montages during sleep, 10 sec per page, amplitude 100 mcV. Normal background activity (vertex waves shown) with activation of epileptiform abnormalities during sleep typical of SeLECTS. Individual 4: EEG showing typical findings in CAE. (E) Bipolar longitudinal montage during wakefulness, 15 sec per page, amplitude 100 mcV. Normal background activity. (F) Bipolar longitudinal montage during wakefulness, 15 sec per page, amplitude 150 mcV. Generalized 3‐Hz spike–wave discharge, 9 sec in duration.

The mean age at the first seizure among the individuals with epilepsy was 9.3 years (SD: 3.1), with ages ranging from 4 to 12 years. Three individuals presented with generalized epilepsy, and three had focal epilepsy. According to the latest ILAE classification and definition of epilepsy syndromes,^22^, ^23^, ^24^ two of the three individuals with generalized epilepsy were diagnosed with Childhood Absence Epilepsy (CAE), and one had Epilepsy with Generalized Tonic–Clonic Seizures Alone (GTCA). Meanwhile, among the three individuals with focal epilepsy, two displayed criteria of Self‐Limited Epilepsy with Centrotemporal Spikes (SeLECTS), previously known as Childhood Epilepsy with Centrotemporal Spikes or Rolandic Epilepsy, and the third was diagnosed with Childhood Occipital Visual Epilepsy (COVE), formerly known as Late‐Onset Benign Occipital Epilepsy or Idiopathic Childhood Occipital Epilepsy–Gastaut Type. None of these individuals had a family history of epilepsy. The main epilepsy features are summarized in Table 1, and selected illustrative EEG studies are shown in Figure 2.

**Table 1:** Individual epilepsy features of the six individuals with dystrophinopathy and epilepsy.

	DMD variant	Age at last visit (y)	Seizure onset (y)	Epilepsy syndrome	Seizure type(s)	EEG	Previous AED(s)/current AED(s)	Epilepsy course (Brodie class)
Ind 1 BMD	Del 45–47	14	9	SeLECTS	Focal onset motor seizures evolving to bilateral tonic–clonic seizures	Normal background activity Multifocal bilateral centrotemporal epileptiform abnormalities with activation during sleep	VPA/–	B
Ind 2 DMD	c.265‐2A>C (intron 11)	11	8	COVE	Focal onset nonmotor seizures without impaired awareness, sensory	Normal background activity Multifocal bilateral centro‐parietal and right posterior temporal epileptiform abnormalities with activation during sleep	VPA/–	A
Ind 3 DMD	c.194delAAAA; p.Lys66Aspfs*8 (exón 4)	19	11	GTCA	Generalized onset motor seizures, tonic–clonic	Normal background activity No epileptiform abnormalities	VPA/VPA	A
Ind 4 DMD	Del 3–11	15	9	CAE	Generalized onset nonmotor seizures (absences) Generalized onset motor seizure, tonic–clonic	Normal background activity Interictal generalized spike–wave discharges at 3 Hz	VPA/LEV	B
Ind 5 BMD	c.9958C>T; p.Pro3320Ser (exón 68)	20	3	SeLECTS	Focal onset motor seizures evolving to bilateral tonic–clonic seizures	Normal or slightly slow background activity Multifocal bilateral centrotemporal epileptiform abnormalities and diffuse bilateral discharges, with activation during sleep	VPA/−	B
Ind 6 DMD	Del 49–50	19	10	CAE	Generalized onset nonmotor seizures (absences)	Normal background activity Interictal generalized spike–wave discharges at 3 Hz. Typical absence seizures during hyperventilation	VPA/VPA	A

Refer to Methods for definitions of Brodie class (A), (B), (C), and (D).

Abbreviations: AED, antiepileptic drug; BMD, Becker muscular dystrophy; CAE, Childhood Absence Epilepsy; COVE, Childhood Occipital Visual Epilepsy; DMD, Duchenne muscular dystrophy; EEG, electroencephalography; GTCA, Epilepsy with Generalized Tonic–Clonic Seizures; LEV, levetiracetam; SeLECTS, Self‐Limited Epilepsy with Centrotemporal Spikes; Sz, seizure; VPA, valproic acid; y, years.

Notably, all six individuals with epilepsy achieved seizure freedom with the initial antiseizure medication used, thus falling into either category “A” (50%) or “B” (50%) according to Brodie's classification, which is detailed in the Methods section. Valproic acid was the initial medication used in all six individuals, showing good control of seizures and overall tolerability. However, one individual experienced weight gain as a side effect of valproic acid, leading to a substitution with levetiracetam. The fact that valproic acid was used in all six cases was decided individually by the neurologists following those individuals and is not due to a specific indication of valproic acid in individuals with dystrophinopathies.

## Discussion

Despite the importance of understanding the prevalence of epilepsy among individuals with dystrophinopathy, only a limited number of studies have looked at this in detail and even fewer have explored its clinical and electroencephalographic features.^6^, ^7^, ^8^, ^9^, ^10^, ^11^ In an effort to address this gap, our study analyzed a cohort of 416 individuals diagnosed with dystrophinopathy.

In contrast to prior reports indicating an approximate prevalence of around 5%, ranging from 3.1% and 7.9%, our data suggest that the prevalence of epilepsy in individuals with dystrophinopathy is lower (1.4%; 95% confidence interval: 0.7–3.2%). Nonetheless, it is important to note that this prevalence still surpasses the prevalence of epilepsy in the general population, estimated at 0.5–1.0% in children and adolescents and 0.5–1.6% in the total population.^7^, ^25^, ^26^ The observed lower prevalence in our large cohort, the largest of its kind to date, could potentially be attributed to random variation, as individuals with epilepsy cases are relatively scarce in all the cohorts and minor differences can result in significant percentage variations. An alternative explanation may be a selection bias in other series, as individuals without epilepsy may be more likely to be excluded from the cohort, whereas it is unlikely for someone with epilepsy to be omitted from an epilepsy‐focused study. To mitigate this potential bias, we conducted a systematic review, encompassing all individuals with dystrophinopathy followed over a specific period of years within each of the three participating centers.

The most prevalent types of epilepsy in our cohort mirrored those commonly observed in the broader pediatric population. Encouragingly, the majority of epilepsies were effectively controlled with a single antiepileptic drug.

We found no statistically significant difference in epilepsy prevalence between individuals with Duchenne muscular dystrophy and those with Becker muscular dystrophy. Although a trend toward higher epilepsy prevalence in individuals with cognitive impairment was apparent, our findings did not reach statistical significance. Consistent with previous studies,^19^, ^27^, ^28^, ^29^, ^30^ individuals with DMD variants affecting all the brain isoforms of dystrophin were significantly more likely to have intellectual disability or poor academic performance compared to other groups.

Among the limitations of our study, despite the size of the cohort, was the limited number of individuals with epilepsy. Therefore, it is desirable to expand the study to other countries to confirm these findings and assess potential variability in prevalence across different geographical areas. Additionally, our study could not incorporate neuroradiological imaging analysis due to the unavailability of such data for the majority of individuals. Lastly, and although a correlation with epilepsy is not expected, no analysis was conducted on the findings of muscle biopsies either.

In summary, our study challenges the previously accepted prevalence of epilepsy in dystrophinopathies, highlighting that earlier estimates based on small patient cohorts and longstanding assumptions may not accurately depict reality. We suggest that the true prevalence of epilepsy in dystrophinopathies could be substantially lower, possibly half or even one‐third of those earlier figures. By providing valuable insights into the prevalence and characteristics of epilepsy in individuals with dystrophinopathy, our research illuminates the impact of epilepsy within this population. These findings have implications for medical care, particularly for individuals with both dystrophinopathy and epilepsy. Enhanced comprehension of the nuanced aspects of epilepsy in individuals with dystrophinopathy will play a pivotal role in tailored medical management, facilitating more targeted interventions and optimizing the overall care for these individuals.

## Author Contributions

Jesus Alfonso Armijo Gómez, Andres Nascimento, and Dr Daniel Natera‐de Benito conceptualized and designed the study, coordinated and supervised data collection, carried out the initial analyses, drafted the initial manuscript, and critically reviewed and revised the manuscript. Miguel A Fernandez‐Garcia, Ana Camacho, Miguel Lafuente‐Hidalgo, Laura Toledo Bravo‐de Laguna, Laura Carrera‐García, and Jessica Expósito‐Escudero collected data and critically reviewed and revised the manuscript. Marlin Liz and Jana Domínguez‐Carral supervised data collection of epilepsy, carried out the initial analyses, and critically reviewed and revised the manuscript. Berta Estévez‐Arias supervised data collection of genetics, carried out the initial analyses, and critically reviewed and revised the manuscript. All authors approved the final manuscript as submitted and agree to be accountable for all aspects of the work.

## Funding Information

This study has been funded by Instituto de Salud Carlos III (ISCIII) through the project “CP22/00141” and co‐funded by the European Union CP22/00141.

## Conflicts of Interest

None of the authors has any conflict of interest to disclose.

## Data Availability

Any data not published within the article will be shared from the corresponding author, upon reasonable request.
